# Association between oxygenation and 6-month mortality during post-cardiac arrest care

**DOI:** 10.1186/cc12253

**Published:** 2013-03-19

**Authors:** M Skrifvars, J Vaahersalo, M Reinikainen, S Bendel, J Kurola, M Tiainen, R Raj, V Pettilä, T Varpula, FinnResusci Study Group

**Affiliations:** 1 Helsinki University Hospital, HUS, Finland; 2 North Karelia Central Hosital, Joensuu, Finland; 3 Kuopio University Hospital, Kuopio, Finland

## Introduction

Optimal oxygenation level during post-cardiac arrest (PCA) care is currently undefined, and studies have suggested harm from hyperoxia exposure [1]. We aimed to assess the optimal oxygenation level and possible associations of time-weighted exposure to hyperoxia on outcome in patients during PCA care.

## Methods

We conducted a prospective observational cohort study in 21 ICUs in Finland between 2009 and 2010. The Utstein Guidelines were used for collecting resuscitation and PCA care data, such as initial rhythm and delay to return of spontaneous circulation (ROSC). Measured arterial blood oxygen values during the first 24 hours from admission to the ICU were divided into the following predefined oxygenation categories: low (<10 kPa), normal (10 to 19 kPa), intermediate (20 to 29 kPa), and high (>30 kPa). Exposure to hyperoxia was defined as paO_2 _levels higher than 40 kPa [1]. Time spent in different oxygenation categories and the highest, lowest and median oxygen values during the first 24 hours were calculated and included in separate multivariate regression models along with age, delay to ROSC, initial rhythm and the use of therapeutic hypothermia for the prediction of 6-month mortality.

## Results

A total of 489 patients were included. The average number of paO_2 _measurements during the first 24 hours was eight per patient. A total of 6% of patients experienced paO_2 _values higher than 40 kPa at any time during the first 24 hours. Average times spent in each time oxygenation category during the first 24 hours were as follows: low 14%, normal 69%, intermediate 14%, and high 2% of the time. Survivors spent less time in the low band (*P *= 0.029) and more time in the intermediate band (*P *= 0.029) compared with nonsurvivors. The median paO_2 _during the first 24 hours was higher in survivors than in nonsurvivors (15 kPa vs. 14 kPa, *P *= 0.016) but there was no difference in lowest (11 kPa vs. 10 kPa, *P *= 0.162) or the highest paO_2 _values (22 kPa vs. 20 kPa, *P *= 0.054). In separate multivariate models neither time spent in the low or the intermediate categories, or the median, highest or lowest paO_2 _was found to correlate with mortality.

## Conclusion

In this multicentre observational study we were unable to define an optimal oxygenation level during PCA care, but hypoxia seemed to be more harmful than hyperoxia. Exposure to hyperoxia was less common than in previous trials, and we were unable to confirm previous findings indicating an association with mortality.
